# The association between BMI, smoking, drinking and thyroid disease: a cross-sectional study in Wuhan, China

**DOI:** 10.1186/s12902-021-00852-0

**Published:** 2021-09-13

**Authors:** Xiao Chen, Juan-juan Wang, Li Yu, Han-yu Wang, Hui Sun

**Affiliations:** 1 Department of Endocrinology, Union Hospital, Tongji Medical College, Huazhong University of Science and Technology, 1277 JieFang Avenue, 430022 Wuhan, Hubei China; 2 Department of Endocrinology, The Central Hospital of Wuhan, 430022 Wuhan, China; 3 Department of Emergency, Union Hospital, Tongji Medical College, Huazhong University of Science and Technology, 430022 Wuhan, China

**Keywords:** Hypothyroidism, Thyroid nodules, BMI, Smoking, Drinking, Obesity

## Abstract

**Background:**

There is no clear conclusion on the relationship between thyroid disease and obesity and lifestyle factors such as smoking and drinking. In this study, we analysed the association of body mass index (BMI), smoking and drinking with subclinical hypothyroidism (SHO) and thyroid nodules (TNs) with the results of a cross-sectional survey of urban residents in central China and discussed the potential mechanism linking these predictive factors and the two diseases.

**Methods:**

This study included 1279 participants who were recruited from a Chinese community in 2011 and 2012. A questionnaire, laboratory examination and ultrasound diagnosis were conducted on these participants. Binary logistic regression analysis was used to analyse these factors.

**Results:**

Overweight (BMI ≥ 25 kg/m^2^) was closely related to SHO and TNs in univariate and multivariate logistic regression analyses. Smoking had a protective effect on SHO and TNs, while drinking had a protective effect on TNs in univariate logistic regression and multivariate logistic regression with some covariates, but there was no significant difference between smoking and drinking and the two kinds of thyroid diseases in multivariate logistic regression analysis with all the covariates. In subgroup analysis, BMI ≥ 25 kg/m2 was significantly associated with SHO in people with positive thyroid antibodies (odds ratio (OR) = 2.221, 95 % confidence interval (CI): 1.168–4.184, *P* = 0.015) and smokers (OR = 2.179, 95 % CI: 1.041–4.561, *P* = 0.039). BMI ≥ 25 kg/m2 was significantly associated with TNs in people over 60 years old (OR = 2.069, 95 % CI: 1.149–3.724, *P* = 0.015) and drinkers (OR = 3.065, 95 % CI: 1.413–6.648, *P* = 0.005). Drinking alcohol had a protective effect on TNs in smokers (OR = 0.456, 95 % CI: 0.240–0.865, *P* = 0.016) and people with BMI ≥ 25 kg/m^2^ (OR = 0.467, 95 % CI: 0.236–0.925, *P* = 0.029). No significant association was found between smoking and the two thyroid diseases in different subgroups.

**Conclusions:**

Obesity is a risk factor for both TNs and SHO, especially in elderly individuals and people with positive thyroid autoantibodies. Obesity and metabolic syndrome may be more associated with TNs than SHO. Smoking may have a protective effect on thyroid disease, while drinking may have a protective effect only on TNs.

**Supplementary Information:**

The online version contains supplementary material available at 10.1186/s12902-021-00852-0.

## Background

At present, thyroid nodules (TNs) and subclinical hypothyroidism (SHO) are very common thyroid diseases worldwide. Studies have reported that the prevalence of SHO is 4–20 % [1]. In addition, with improvements in imaging diagnosis, the prevalence of TNs has increased in recent years [2]. Because of the occult symptoms, these two thyroid diseases are often ignored by most patients, and the detection of the disease mainly depends on screening in the general population. However, a number of studies have shown that SHO is associated with an increased risk of a variety of cardiovascular diseases [3] and cognitive impairment [4] and may develop into dominant hypothyroidism. Additionally, solitary TNs have varying degrees of the risk of progression [5]. Therefore, the early diagnosis and early symptoms of these two diseases, especially in susceptible populations with different risk factors, is of great significance.

Numerous studies on risk factors related to SHO and TNs have been performed, and sex and age are known to increase the risk of thyroid disease [6–8]. Hashimoto’s thyroiditis also increases this risk. However, there is no clear conclusion on adverse metabolic conditions such as obesity and lifestyle habits such as smoking and drinking. Although some studies have found that metabolic syndrome and obesity can lead to an increased risk of thyroid disease [9–11], this conclusion may have different manifestations in people with different characteristics, such as patients with autoimmune thyroid diseases and different ages and sexes, and there are no studies on concurrent SHO and TNs. Considering that there may be some differences and connections in the pathogenesis of these two thyroid diseases, the simultaneous study of predictors of these two diseases could help us to better understand and further explore the potential association between thyroid function and anatomy. In this study, by further evaluating the correlation between obesity and lifestyle and the two diseases in this subgroup, we can eliminate the influence of a variety of confounding factors to a certain extent, which is helpful for determining the influence of obesity, smoking and drinking on the physiology and morphology of the thyroid gland. For people with varying degrees of disease risk, this information may help to more accurately guide thyroid examination and early intervention.

In our study, thyroid function and thyroid ultrasound examinations were performed on 1500 randomly screened healthy residents of Wuhan, China. The effects of obesity, smoking and drinking on the prevalence of SHO and TNs were evaluated. After adjusting for the related confounding factors with subgroup analysis, we further compared whether these factors played a more significant role in thyroid disease in people with specific demographics.

## Methods

### Study participants

This study was conducted in a community in Wuhan, China, and recruited a total of 1500 healthy residents in 2011 and 2012. The study participants were a part of a cross-sectional survey of thyroid disease in 10 cities across China. After a comprehensive assessment of factors such as GDP(Gross National Product) per capita, concentration of commercial resources, resident vitality, lifestyle diversity, and future development potential and considering that the study subjects should preferably choose residents living in the city for more than 5 years, this community was chosen as the most epidemiologically regionally representative place to perform the study. Recruitment was performed by using stratified cluster sampling. The methods to control bias included three points: (1) Respondents were recruited in the order of community population registration. (2) Not considering respondents’ requests to participate in the study to prevent a false increase in prevalence caused by a tendency to seek medical care. Thus, the recruitment process was done in the community instead of the hospital. (3) The age ratio and sex ratio of the respondents were verified. All subjects provided informed consent for inclusion before participating in the study. The study was conducted in accordance with the Declaration of Helsinki, and the protocol was approved by the Ethics Committee of the Medical Ethics Committee of China Medical University (Serial number: IRB[2008]34). The inclusion criteria were as follows: (1) over the age of 20 years, (2) Han nationality, (3) living in Wuhan for at least 10 years, (4) did not receive iodine-containing contrast agent or drugs such as amiodarone within the past three months, and (5) no previous thyroid disease. We excluded subjects with clinically abnormal thyroid function, missing important information and pregnant and lactating women. After screening participants who met the study criteria, participants were divided into an SHO group (N = 194), a non-SHO group (N = 1085), a TN group (N = 238) and a non-TN group (N = 1041) according to the diagnostic criteria.

### Data collection

A standard questionnaire was designed to acquire basic information on participants, including name, sex, ethnicity, history of smoking and alcoholism, eating habits, medication history, female reproductive history, and family and personal history of thyroid disorders. According to the questionnaire results, laboratory and ultrasound examinations were carried out on all the subjects who met the study criteria. Spot urine and blood samples were collected in the morning after overnight fasting. Urine iodine was determined by the ammonium persulfate method based on the Sandell-Kolthoff reaction (T6 UV spectrophotometry). Concentrations of serum thyroid-stimulating hormone (TSH), free triiodothyronine (FT3), free tetraiodothyronine (FT4), thyroperoxidase antibody (TPOAb), and thyroglobulin antibody (TgAb) were determined by an electrochemiluminescence immunoassay (Roche Kit, Cobas-e601 analyzer). Thyroid ultrasonography was performed by professional physicians using a 7.5-MHz transducer (GE, LOGIQ a100). The normal reference values were as follows: FT4 12.0–22.0 pmol/L, FT3 3.1–6.8 pmol/L, TSH 0.27–4.2 mU/L, TPOAb 0–34 IU/mL, and TgAb 0-115 IU/mL. The height (measured to the nearest 0.1 cm) and weight (measured to the nearest 0.1 kg) of participants was also measured while the individuals wore light clothes without shoes. Their diastolic blood pressure (DBP) and systolic blood pressure (SBP) (measured to the nearest 1 mmHg) were measured using an electronic sphygmomanometer (HBP-9020, Omron, Japan) when the individuals were seated and quiet; two measurements were taken at an interval of 30 s between each measurement, and the results were averaged [12].

### Definition of variables

In this study, the definition of TNs was based on the American Thyroid Association guidelines [13]. A TN was defined as a small lesion differentiated from the rest of the thyroid parenchyma, possessing a solid part, with or without the presence of a cystic part. SHO was defined as serum FT3 and FT4 within the normal range but TSH exceeding 4.2 mU/L. TPOAb positivity was defined as serum TPOAb of more than 34 IU/L, and TgAb positivity was defined as serum TgAb of more than 115 IU/L. The BMI index adopted the WHO standards [14], in which weight was divided by the square of height, and a result greater than 25 kg/m^2^ was defined as overweight. Smokers includes people who currently smoked or had ever smoked, and the definition of drinkers was similar to that of smokers, including people who have or have had drinking habits. The specific amount or types of smoking and drinking were not accurately investigated in this study.

### Statistical analysis

All statistical analyses were conducted with SPSS software (IBM, Armonk, NY, USA, version 26.0). We first performed a differential analysis of the baseline data of the population. Then, we conducted correlation analysis for variables that showed significant differences in the difference analysis, and used univariate logistic regression and multivariate logistic regression. However, blood pressure and waistline were not included in correlation analysis, as their uneven distribution in the population would have made it difficult to convert continuous variables into categorical variables. Additionally, the collinear effect between the variables and BMI may have affected the performance of the model. When building multivariate regression models, all independent variables that were significant in univariate analysis were used as covariates. Then, a method was employed to change the modelling strategy by adding covariates one by one to perform sensitivity analysis of variables that were not statistically significant in the multivariate regression analysis. Finally, we conducted subgroup analyses with BMI, smoking, and alcohol consumption as study factors to further assess possible interactions between other predictors and them. The Kolmogorov-Smirnov test was used to test normality. Normally distributed data are expressed as the mean ± standard deviation (SD), and abnormally distributed data are expressed as the median and interquartile range. The t test was used to compare normal and continuous variables, the nonparametric rank test (Kruskal-Wallis) for non-normal and continuous variables, and the chi-square test to compare the prevalence of thyroid disorders between different groups.Odds ratios (ORs) and 95 % confidence intervals (95 % CIs) were calculated using binary logistic regression analyses. Multivariate logistic regression was used to analyse the association between BMI and smoking, alcohol and disease in subgroup analysis. A two-sided *P* < 0.05 was considered significant.

## Results

### Baseline characteristics

**Fig. 1:**
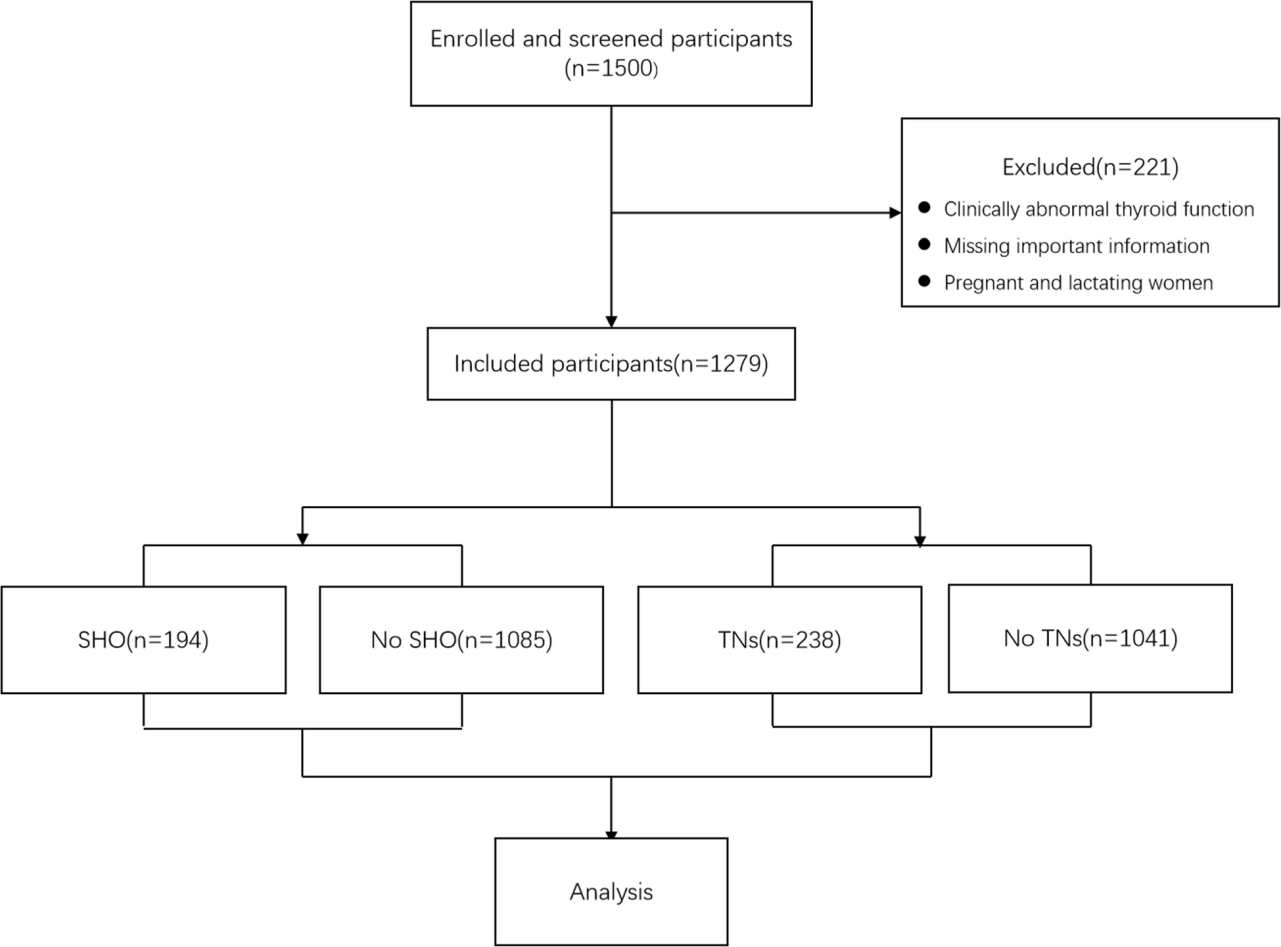
Flow chart of enrolment and grouping for SHO, and TNs. Among a total of 1500 subjects, 221 were excluded according to the criteria, and the remaining 1279 subjects were divided into the study group and control group according to the disease and non-disease status for TNs and SHO. Statistical analysis was performed in the respective study group and control group

After excluding individuals who did not meet the study criteria and were missing information such as thyroid function, BMI and other key indicators, a total of 1279 of the 1500 participants were included in the study. There were 194 patients with SHO and 238 patients with TNs. These patients were compared with 1045 patients with non-SHO and 1081 patients without TNs (Fig. 1). In Table 1, the baseline data of the corresponding populations with the two diseases and their respective control groups are shown. The prevalence of both TNs and SHO was significantly higher in overweight people (22.8 % vs. 16.0 %, P = 0.003 & 18.1 % vs. 13.4 %, *P* = 0.023) and non-smokers (20.1 % vs. 15.1 %, P = 0.038 & 17.3 % vs. 10.1 %, P = 0.001) than in the corresponding controls. However, only the prevalence of TNs was significantly higher in drinkers than in non-drinkers (14.1 % vs. 19.7 %, P = 0.037). In addition, the baseline data of patients with the two diseases, such as sex, age, positive thyroid antibody, blood pressure level and waist circumference, were also different from those of healthy people. In particular, blood pressure (120.54 ± 20.01 vs. 113.93 ± 19.83 mmHg, P = 0.000 & 77.15 ± 11.35 vs. 75.09 ± 11.9 mmHg, P = 0.015) and waist circumference (84 vs. 82 cm, *P* = 0.000) associated with poor metabolism were higher in the population with TNs, while there was no such trend in people with SHO. Urinary iodine levels were not significantly different in people with both thyroid diseases compared with healthy people, whether evaluated as continuous or categorical variables.

**Table 1:** Baseline characteristics of the participants with or without SHO and TNs

Characteristics	SHO (*N* = 194)	No SHO (*n* = 1085)	P	TN (*n* = 238)	No TN (*n* = 1041)	P
Sex (n, %)			0.000			0.004
Male	46(8.8 %)	478(91.2 %)		78(14.9 %)	446(85.1 %)	
Female	148(19.6 %)	607(80.4 %)		160(21.2 %)	595(78.8 %)	
Age (years)	47.25 ± 14.99	44.57 ± 14.54	0.021	52.84 ± 14.65	43.17 ± 14.43	0.000
Waistline, median (IQR), cm	84(76–90)	82.00(74–88)	0.098	84.10(77–92)	82(74–88)	0.000
SBP (mmHg)	117.33 ± 20.52	114.78 ± 20.14	0.105	120.54 ± 20.01	113.93 ± 19.83	0.000
DBP (mmHg)	76.41 ± 11.30	75.30 ± 11.91	0.229	77.15 ± 11.35	75.09 ± 11.91	0.015
TPOAb, n (%), IU/L			0.000			0.647
> 34	38(26.2 %)	107(73.8 %)		29(20.0 %)	116(80.0 %)	
≤ 34	156(13.8 %)	978(86.2 %)		209(18.4 %)	925(81.6 %)	
TgAb, n (%), IU/L			0.000			0.982
> 115	45(29.8 %)	106(70.2 %)		28(18.5 %)	123(81.5 %)	
≤ 115	149(13.2 %)	979(86.8 %)		210(18.6 %)	918(81.4 %)	
Smoker (n, %)			0.001			0.038
Yes	38(10.1 %)	339(89.9 %)		57(15.1 %)	320(84.9 %)	
No	156(17.3 %)	746(82.7 %)		181(20.1 %)	721(79.9 %)	
Alcoholic (n, %)			0.127			0.037
Yes	31(12.1 %)	225(87.9 %)		36(14.1 %)	220(85.9 %)	
No	163(15.9 %)	860(84.1 %)		202(19.7 %)	821(80.3 %)	
Fatty liver (n, %)			0.052			0.053
Yes	60(18.5 %)	264(81.5 %)		72(22.2 %)	252(77.8 %)	
No	134(14.0 %)	821(86.0 %)		166(17.4 %)	789(82.6 %)	
BMI, n (%), kg/m²			0.023			0.003
≥ 25	88(18.1 %)	399(81.9 %)		111(22.8 %)	376(77.2 %)	
< 25	106(13.4 %)	686(86.6 %)		127(16.0 %)	665(84.0 %)	
Urine iodine, median (IQR), µg/L	210(151–298)	222(154–305)	0.718	224(150–331)	219(154–303)	0.557
Urine iodine, n (%), µg/L			0.737			0.783
≤ 200	84(15.6 %)	453(84.4 %)		96(17.9 %)	441(82.1 %)	
200–300	63(15.6 %)	340(84.4 %)		75(18.6 %)	328(81.4 %)	
> 300	47(13.9 %)	292(86.1 %)		67(19.8 %)	272(80.2 %)	

Abbreviations: *SHO* subclinical hypothyroidism, *TNs* thyroid nodules, *N* number, *IQR* interquartile range, *BMI* body mass index, *SBP* systolic blood pressure, *DBP* diastolic blood pressure, *TPOAb* thyroid peroxidase antibody, *TgAb* thyroglobulin antibody

### Association between different risk factors and the two thyroid diseases

Table S1 further describes the significant association between different predictive factors and thyroid disease in the difference analysis (see Supplementary Tables 1, Additional File 1). After adjusting for other covariates, multivariate logistic regression analysis showed that age, sex and BMI were all closely related to the two thyroid diseases, and thyroid antibody positivity was closely related to SHO. After further analysis with age as a classification variable, people in the 40–60 year age group had the highest risk of SHO (OR = 1.497, 95 % CI: 1.052–2.129, *P* = 0.025), while people older than 60 years old had the highest risk of TNs (OR = 4.459, 95 % CI: 2.973–6.687, *P* = 0.000). Among patients with thyroid antibody positivity, patients with single-positive TPOAb had the highest risk of SHO (OR = 2.329, 95 % CI: 1.345–4.032, *P* = 0.003). In univariate logistic regression analysis, smoking had protective effects on TNs (OR = 0.710, 95 % CI: 0.512–0.983, *P* = 0.039) and SHO (OR = 0.536 95 % CI: 0.368–0.782, *P* = 0.001), and drinking had a protective effect on TNs (OR = 0.665, 95 % CI: 0.453–0.977, *P* = 0.038). However, in multivariate logistic regression analysis, the protective effects of smoking and alcohol consumption did not remain significant. Table 2 shows the change in the protective effect of smoking and alcohol consumption on both thyroid diseases when the included covariates were changed one by one in multivariate regression analysis. This protective effect was significant when the included covariates did not include sex or did not include both smoking and drinking.

**Table 2:** Effects of smoking and drinking on SHO and TNs in different modelling modalities

	OR (95 % CI)					
	SHO		TNs			
	non-smoker	smoker	non-smoker	smoker	non-alcoholic	alcoholic
Model 1: no adjustment	reference	0.536(0.368–0.782)	reference	0.710(0.512–0.983)	reference	0.665(0.453–0.977)
*p* Values	0.001		0.039		0.038	
Model 2: adjusting for BMI	reference	0.496(0.339–0.728)	reference	0.652(0.469–0.908)	reference	0.644(0.438–0.948)
*p* Values	0.001		0.014		0.001	
Model 3: adjusting for BMI and age	reference	0.487(0.332–0.715)	reference	0.656(0.469–0.919)	reference	0.599(0.404–0.888)
*p* Values	0.001		0.011		0.011	
Model 4: adjusting for BMI, age, and antibody	reference	0.549(0.372–0.810)	N/A		N/A	
*p* Values	0.001					
Model 5: adjusting for BMI, age, antibody, and sex	reference	1.002(0.596–1.685)	N/A		N/A	
*p* Values	0.993					
Model 6: adjusting for BMI, age, sex, and alcohol	N/A		reference	0.928(0.592–1.454)	N/A	
*p* Values				0.745		
Model 7: adjusting for BMI, age, sex, and smoking	N/A		N/A		reference	0.744(0.479–1.156)
*p* Values					0.189	

Abbreviations: *SHO* subclinical hypothyroidism, *TNs* thyroid nodules, *BMI* body mass index. All models were developed by binary logistic regression

### Subgroup analysis of BMI, smoking and drinking

Table 3 and Table S2 present correlation analysis with BMI, smoking and drinking as independent predictive factors for the two thyroid diseases in different subgroups (see Supplementary Tables 2 and Additional File 2). After adjusting for other covariates, BMI ≥ 25 kg/m2 was significantly associated with SHO in people with positive thyroid antibodies (OR = 2.221, 95 % CI: 1.168–4.184, P = 0.015) and smokers (OR = 2.179, 95 % CI: 1.041–4.561, P = 0.039), and it was significantly associated with TNs in people over 60 years old (OR = 2.069, 95 % CI: 1.149–3.724, P = 0.015) and drinkers (OR = 3.065, 95 % CI: 1.413–6.648, P = 0.005). Drinking alcohol had a protective effect on TNs in smokers (OR = 0.456, 95 % CI: 0.240–0.865, P = 0.016) and people with BMI ≥ 25 kg/m2 (OR = 0.467, 95 % CI: 0.236–0.925, P = 0.029). No significant association was found between smoking and the two thyroid diseases in different subgroups.

**Table 3:** Associations of SHO with BMI and smoking in subgroups stratified by other variables

Subgroup	N	BMI ≥ 25 kg/m²				Smoker			
		n	OR	95 %CI	*P* value	n	OR	95 %CI	*P* value
Sex^a^									
Male	46	28	1.770	0.945–3.315	0.074	29	0.896	0.470–1.709	0.739
Female	148	60	1.464	0.990–2.164	0.056	9	1.352	0.605–3.023	0.463
Age (years)^b^									
≤ 40	66	24	1.562	0.880–2.773	0.128	15	1.045	0.459–2.381	0.916
40–60	94	41	1.368	0.850–2.202	0.197	18	1.293	0.574–2.914	0.536
> 60	34	23	2.075	0.939–4.585	0.071	5	0.678	0.199–2.308	0.535
Antibody^c^									
Antibody -	134	57	1.436	0.976–2.114	0.066	26	0.763	0.419–1.389	0.376
Antibody +	60	31	2.221	1.168–4.184	0.015	12	2.757	0.909–8.359	0.073
Smoker^d^									
No	156	24	1.443	0.994–2.096	0.054	0	N/A		
Yes	38	64	2.179	1.041–4.561	0.039	38	N/A		
BMI (kg/m²)^e^									
< 25	106	0	N/A			24	0.833	0.382–1.816	0.645
≥ 25	88	88	N/A			14	1.224	0.597–2.512	0.581

Abbreviations: *BMI* body mass index, Antibody + (positive) for at least one thyroid antibody, Antibody - negative for both thyroid antibodies. All the variables were analysed by multivariate logistic regression: ^a^ Adjusted for age, antibody, smoking, and BMI. ^b^ Adjusted for sex, antibody, smoking, and BMI. ^c^ Adjusted for sex, age, smoking, and BMI. ^d^ Adjusted for sex, age, antibody, and BMI. ^e^ Adjusted for sex, age, antibody, and smoking

## Discussion

At present, differing results on the relationship between obesity and thyroid disease have been reported. Obesity can cause insulin resistance and hyperinsulinaemia, making the body prone to a state of energy reserve [12]. This may inhibit the synthesis and release of thyroid hormones, and studies have shown that insulin can also promote the abnormal proliferation of thyroid tissue [15, 16]. In addition, the accumulation of fat in the body and the increase in free fatty acids in circulation may also lead to the steatosis and infiltration of the thyroid gland, resulting in changes in thyroid morphology and function [17]. By comparing thyroxine levels of patients before and after bariatric surgery, several studies found that TSH levels significantly decreased after bariatric surgery, which was maintained for long periods without L-T4 therapy, indicating that SHO tends to spontaneously improve after weight loss [18–20]. Since many weight loss operations are performed by gastrectomy, obesity-mediated SHO is more likely to be affected by some intestinal hormones [21]. For example, GLP-1 can improve the activity of deiodinase and promote the synthesis of thyroid hormone, while the secretion of this hormone increases after weight loss [22]. In addition, leptin secreted by adipose tissue has also been shown to affect the regulation of the hypothalamic-pituitary-thyroid axis, thereby inhibiting thyroid hormone release and stimulating TSH secretion [23–25].

In this study, difference and correlation analysis showed that the risk of SHO and TNs in obese patients (BMI ≥ 25 kg/m2) was higher than that in non-obese patients. Other factors associated with obesity and metabolic syndrome, such as waist circumference and blood pressure, however, were only associated with TNs. Two studies from South Korea on the clinical characteristics of SHO and TNs also found similar results [26, 27], although the level of BMI was positively correlated with the level of TSH, and this correlation persists even though the TSH level slightly fluctuates within the normal reference range. Thus, the presence or absence of metabolic syndrome does not affect the occurrence of SHO. This suggests that metabolic status may have a more significant effect on the occurrence of TNs than SHO. To some extent, this proves that there is no direct correlation between insulin resistance and SHO. In addition, a study of women with polycystic ovary syndrome (PCOS) found that other metabolic conditions were associated with TSH levels only in obese women with PCOS [28], suggesting that obesity may be a necessary condition to mediate the mechanism of hyperinsulinaemia on the thyroid gland. In contrast, the relationship between TNs and metabolic disorders, such as impaired glucose tolerance, metabolic syndrome and insulin resistance, has been confirmed in a number of observational studies [29–31]. In our study, the high correlation between TNs and these indexes also suggests that the effect of adverse metabolic status on the thyroid may have a greater effect on non-functional and morphological changes, which do are not necessarily mediated by obesity. This needs to be proven by further pathological results.

Among participants in different age groups, we found that people aged 40–60 years had the highest risk of SHO. However, for TNs, risk increased with age. In elderly patients, the effect of BMI on TNs was also more obvious. The different effects of age on the two diseases may be due to subtle differences in pathogenesis, the exact mechanism of which is still unclear. However, we speculate that the protective effect of advanced age (> 60 years old) on SHO may be due to the decrease in fat factors such as leptin, which can inhibit the secretion of thyroid hormone, in elderly individuals. In contrast, the occurrence of TNs is less affected by the regulation of hormones in the body, while the slow local metabolism of the thyroid gland in elderly individuals leads to the accumulation and proliferation of thyroid tissue, which has a synergistic effect with obesity. In terms of sex, the prevalence of thyroid disease was higher in women than in men, but subgroup analysis did not show that BMI had different effects on men and women. Our findings may be due to the small sample size, but the association among sex, obesity and thyroid disease may be complex. A previous study, also from Tianjin, China, showed that BMI was not associated with the incidence of SHO in women but played a protective role in men [32], which contradicted the results of many previous studies [33–35]. Oestrogen may have a unique pathological effect on the thyroid gland, making women susceptible to thyroid disease [36], but it also has a different effect on the distribution and metabolism of human fat in women than in men. These two mechanisms drive the different relationship between obesity and thyroid disease in different sexes, and more in-depth research is needed to prove this.

Thyroid autoantibody levels are increased in patients with SHO, but there is no such trend in patients with TNs. Patients with TPOAb have the highest risk of SHO because TPOAb has been shown to play a direct role in thyroid destruction through antibody- and complement-mediated cytotoxicity. In the subgroup analysis, BMI was directly associated with the risk of SHO only in antibody-positive patients. There is often abnormal lymphocyte infiltration in the thyroid tissue of these patients, and studies have shown that obesity itself is also a chronic inflammatory state. In obese patients, there is a trend of increased cytokine secretion by immune cells as inflammatory markers [37]. By comparing the changes in inflammatory factors in patients with SHO after weight loss, Zhu, C et al. found that the decreasing trend of pro-inflammatory factors such as IL-6, CRP and TNF-α was positively correlated with the decrease in TSH [20]. This suggests that obesity and Hashimoto’s thyroiditis may have a synergistic effect on thyroid tissue damage, making the effect of obesity on the thyroid more obvious. Hashimoto’s thyroiditis patients with obesity are more likely to develop subclinical or even clinical hypothyroidism than non-obese patients.

Recent studies have shown that lifestyle factors such as smoking and drinking have certain benefits for thyroid disease, but the exact mechanism is unknown. A cohort study of 10 million people in South Korea showed that smokers and drinkers had a reduced risk of thyroid cancer, and the two had a synergistic effect [38]. Other studies have shown that stopping smoking leads to an increased risk of thyroid autoantibodies, which leads to SHO [39]. In our study, the effects of smoking and drinking on thyroid disease showed very complex patterns after multivariate analysis and subgroup analysis. Univariate logistic regression showed that smoking had a certain protective effect on the two kinds of thyroid diseases, and the prevalence rate of the two kinds of thyroid diseases in smokers was significantly lower than that in non-smokers. However, after adjusting for other covariates, the protective effect of smoking was eliminated. It is worth mentioning that when we changed multiple modelling strategies and found that the protective effects of smoking and alcohol were significant when the covariate sex was excluded from the model or when smoking and drinking were not included in the model together for analysis. This may be because the number of smokers and drinkers varies too much among sexes, especially the number of women who smoke and drink is too small, and there were collinear effects between smoking and drinking and sex. However, in the subgroup analysis, the effect of obesity on thyroid disease in people of different sexes and the effect of obesity on thyroid disease in smokers and non-smokers and drinkers and non-drinkers are not consistent. This suggests that the effects of smoking and alcohol consumption are not completely countered by sex. Therefore, the protective effect of smoking and alcohol consumption on thyroid disease deserves further study.

It is worth noting that in the subgroup analysis, smokers were more likely to be affected by BMI-induced SHO than non-smokers after adjusting for other factors. Similarly, drinkers are more susceptible to the effects of BMI on TNs than non-drinkers. This may be due to our small sample size because there were only 38 patients in the subgroup of smoking with SHO and 36 patients with TNs who drank alcohol. Considering that the lifestyle factors of smoking and drinking can also lead to obesity and metabolic disorders, the proportion of obese patients is relatively high. Previous studies have also shown that there is no difference in the effects of obese SHO in smokers and non-smokers [40]. However, the independent protective effect of smoking and drinking on thyroid disease seems to contradict the harm caused by obesity. Especially in the subgroup analysis, a history of alcohol addiction had an obvious protective effect on TNs in non-obese people. Therefore, future research on the risk of thyroid disease should pay more attention to people who are obese who also smoke and drink.

In our study, urinary iodine levels were used as continuous variables and numerical variables, but no differences were found between patients with SHO and TNs and healthy individuals. This shows that the iodine nutrition status does not affect the prevalence of thyroid diseases, and the results are not consistent with the results of the national iodine nutrition survey in China [41]. Although it is believed that the effect of iodine on the thyroid occurs in the long-term, the cross-sectional difference in the small sample size may not be significant. However, it also shows that a high-iodine diet as a lifestyle has a less significant effect on the thyroid than obesity, smoking and drinking, and there is no need to deliberately control iodine intake for the prevention of SHO and TNs.

Our study has the following limitations. Because it was based on population screening, the sample size of the disease was small. In addition, the study was a cross-sectional study, so it was not possible to determine the causal relationship between risk factors and diseases. For the subgroups in this study, larger studies are still needed to confirm the exact relationship between the covariates.

## Conclusions

Obesity is a risk factor for thyroid disease, especially in SHO patients with positive thyroid antibodies and in elderly patients with TNs. Compared with SHO, obesity and poor metabolism may have a greater impact on the prevalence of TNs, suggesting that the effect of metabolism on the thyroid is more morphological and non-functional. Smoking may be a protective factor for thyroid disease, while drinking is a protective factor for TNs in obese people. Smoking may have a protective effect on thyroid disease, while drinking may have a protective effect only on TNs. Further studies are needed to confirm these findings.

## Supplementary Information

**Additional file 1.**

**Additional file 2.**

## Data Availability

The datasets used and/or analysed during the current study are available from the corresponding author on reasonable request.

## Abbreviations

WHO: World Health Organization
BMI: Body mass index
DBP: Diastolic blood pressure
ORs: Odds ratios
SBP: Systolic blood pressure
TNs: Thyroid nodules
SHO: Subclinical hypothyroidism
TPOAb: Thyroid peroxidase antibody
TgAb: Thyroglobulin antibody
TSH: Thyroid-stimulating hormone
CRP: C-reactive protein
IL-6: Interleukin-6
PCOS: Polycystic ovary syndrome
FT3: Free triiodothyronine
FT4: Free tetraiodothyronine
GLP-1: Glucagon-like peptide 1
